# Endoplasmic reticulum stress-related genes as prognostic and immunogenic biomarkers in prostate cancer

**DOI:** 10.1186/s40001-024-01818-3

**Published:** 2024-04-20

**Authors:** Lilin Wan, Yunxia Fan, Tiange Wu, Yifan Liu, Ruixin Zhang, Saisai Chen, Chenggui Zhao, Yifeng Xue

**Affiliations:** 1 Southeast University, 87 Dingjia Bridge Hunan Road, Nanjing, 210009 China; 2 Department of Urology, Zhongda Hospital Southeast University, 87 Dingjia Bridge Hunan Road, Nanjing, 210009 China; 3 Department of Laboratory, Zhongda Hospital Southeast University, 87 Dingjia Bridge Hunan Road, Nanjing, 210009 China; 4 Department of Urology, Jintan Affiliated Hospital of Jiangsu University, No.500, Jintan Avenue, Jintan District, Changzhou, 213200 China

**Keywords:** Endoplasmic reticulum stress, Prostate cancer, ERLIN2, CDK5RAP3, Biomarkers

## Abstract

**Background:**

The metastasis and aggressive nature of prostate cancer (PCa) has become a major malignancy related threat that concerns men’s health. The efficacy of immune monotherapy against PCa is questionable due to its lymphocyte-suppressive nature.

**Method:**

Endoplasmic reticulum stress- (ERS-) and PCa-prognosis-related genes were obtained from the Molecular Signatures Database and the Cancer Genome Atlas database. The expression, prognosis and immune infiltration values of key genes were explored by “survival R package”, “rms”, “xCELL algorithm”, and univariate–multivariate Cox and LASSO regression analyses. The “consensus cluster plus R package” was used for cluster analysis.

**Result:**

As ERS-related genes, ERLIN2 and CDK5RAP3 showed significant expressional, prognostic and clinic-pathologic values. They were defined as the key genes significantly correlated with immune infiltration and response. The nomogram was constructed with T-stage and primary treatment outcome, and the risk-prognostic model was constructed in the following way: Riskscore = (− 0.1918) * ERLIN2 + (0.5254) * CDK5RAP3. Subsequently, prognostic subgroups based on key genes classified the high-risk group as a pro-cancer subgroup that had lower mutation rates of critical genes (SPOP and MUC16), multiple low-expression immune-relevant molecules, and differences in macrophages (M1 and M2) expressions. Finally, ERLIN2 as an anti-oncogene and CDK5RAP3 as a pro-oncogene were further confirmed by cell phenotype assays and immunohistochemistry.

**Conclusion:**

We identified ERLIN2 and CDK5RAP3 as ERS-related genes with important prognostic and immunologic values, and classified patients between high- and low-risk subgroups, which provided new prognostic markers, immunotherapeutic targets, and basis for prognostic assessments.

**Supplementary Information:**

The online version contains supplementary material available at 10.1186/s40001-024-01818-3.

## Introduction

Prostate cancer (PCa) has become one of the major malignant tumors that threatens men’s health and survival due to its aggressive metastasis [1]. Previous clinical studies suggest that PCa advances to castration-resistant prostate cancer (CRPC) at 18–24 months after standard androgen deprivation therapy; chances of overall survival (OS) of patients in the long term are difficult to improve despite undergoing chemotherapy and novel endocrine therapies targeting CRPC [2–4]. Due to its treatment-resistant nature and adverse effects, research in the field of PCa immunotherapy, including PD1–PDL1 axis, cytotoxic T lymphocyte antigen (CTLA), and immune checkpoint inhibitors (ICIs), has become a top priority in recent years [5]. Despite obtaining encouraging results with immunologic monotherapy at the preclinical stage, the available outcomes are not convincing when compared with observations recorded in the case of other cancers [5, 6]. Therefore, there is an urgent demand for new immunotherapy-associated targets and prognostic markers to develop desirable immunotherapeutic agents and strategies for PCa.

The tumor-immune microenvironment (TIM) of PCa is an essential area for immunotherapy. Multiple factors could affect alterations in the PCa microenvironment, including endoplasmic reticulum stress (ERS) and periprostatic adipose tissue, ultimately influencing tumor progression and therapy outcomes [8–10, 24]. The TIM of PCa is mostly non-inflammatory and has low neoantigen expression and weak stimulation of the immune system [5]. It is recognized as a lymphocyte-suppressive tumor (also called cold tumor), with the immune system characterized by lymphocyte deficiency and macrophage infiltration [7]. ERS is a protective stress response; the pathological condition is manifested by the accumulation of abnormal proteins in the endoplasmic reticulum (ER) and initiation of the unfolded protein response, which ultimately plays an important role in TIM, oxidative stress, DNA damage, and other activities [8]. Existing studies have found that ERS is closely associated with inflammatory responses regulated by macrophage polarization [8, 9], and interestingly, ERS influences prostate carcinogenesis as well as the progression of castration resistance [10]. Currently, there are no comprehensive bioinformatics analyses exploring the overall role of ERS-related genes in tumor progression and drug resistance in PCa.

**Fig. 1:**
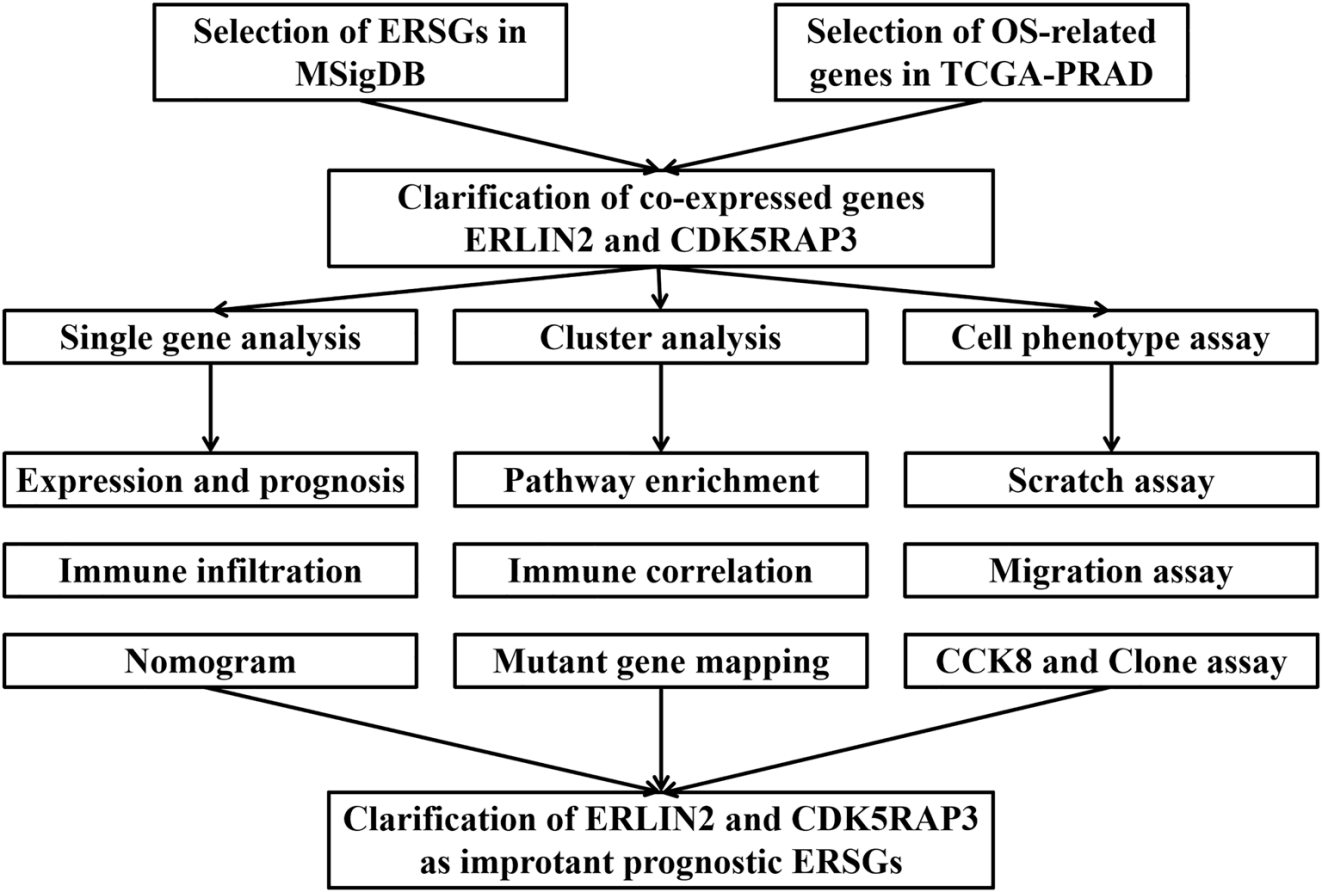
Study design flowchart

The flowchart of this study design is shown in Fig. 1. First, ERS-related genes were screened with the help of Molecular Signatures Database (MSigDB). Prognostic ERS-related genes with important roles were identified in the Cancer Genome Atlas Prostate Adenocarcinoma (TCGA-PRAD) disease cohort, and their correlations with the immune microenvironment, immunotherapy response, and drug resistance were explored. Then they were divided into high- and low-risk subgroups using cluster analysis, and gene expression differences, enrichment pathways, immune infiltration, and immune-related molecular differences among different subgroups were explored. Finally, ERLIN2 as an anti-oncogene and CDK5RAP3 as a pro-oncogene were further confirmed by cell phenotype assays and immunohistochemistry.

## Method

### Data acquisition

In MSigDB (http://www.gsea-msigdb.org/gsea/msigdb/index.jsp), two ERS-related datasets were downloaded (GO RESPONSE TO ENDOPLASMIC RETICULUM STRESS and GO REGULATION OF RESPONSE TO ENDOPLASMIC RETICULUM STRESS), and258 ERS-related genes were ultimately obtained. The RNA-seq dataset and clinic-pathologic information of PCa were downloaded from the TCGA database (https://portal.gdc.cancer.gov/), and 344 PCa-related prognostic OS genes were obtained with the help of the “survival R package”.

### Gene screen

The ERS-related prognostic genes were analyzed by the Venn diagram. Differential expression, OS, progress-free interval (PFI), and correlation with clinic-pathological features of PCa in the TCGA-PRAD cohort were analyzed by the “survival R package”.

### Model construction

Independent risk prognostic factors were clarified by univariate–multivariate Cox regression analysis. A nomogram of common differential genes was constructed with the “rms” package to predict 3-, 5-, and 10-year PFI. Prognostic calibration curves were plotted based on the “survival R package”. Moreover, the risk prognostic model was constructed by LASSO regression analysis and the distributional characteristics of different groups were analyzed. The validity of prognostic indicators was evaluated using the area under the curve (AUC) of the time-dependent ROC curve.

### Cluster analysis

Cluster analysis was performed using the “Consensus Cluster Plus R package”, using 1-Pearson correlation distance for PAM clusters, with 10 repetitions of re-sampling for 80% of the population. The optimal number of clusters was determined using the empirical cumulative distribution function plot. Differences in mRNA expression between subtypes were analyzed using the “limma package”. The screening criteria for mRNA differential expression were adjusted *p* < 0.05 and |fold change| > 1.5. Functional enrichment analyses of key genes and different cluster subgroups were performed using the “cluster Profiler” to explore Gene Ontology (GO) and Kyoto Encyclopedia of Genes and Genomes (KEGG), provided that the *q*- and *p*-values were < 0.05. Furthermore, differences in gene sets across subgroups were further assessed based on the GSEA software (http://www.broadinstitute.org/gsea/index.jsp). PCa somatic mutation data were obtained from the TCGA GDC data portal and waterfall plotted using “maftools”.

### Immune infiltration and response

For single-gene immunoassays, the “xCELL algorithm” was used to clarify the immune infiltration status of key genes in TCGA-PRAD. The “ggplot2” R package was used to analyze the relationship between immune checkpoint-associated genes such as SIGLEC15, TIGIT, CD274, HAVCR2, PDCD1, CTLA4, LAG3, and PDCD1LG2. In the TCGA-PRAD and ICGA-PRAD cohorts, Perl scripts were used to calculate tumor mutation burden (TMB) scores, which were then corrected by the total length of the exons. Potential ICB responds to ICIs using the Tumor Immune Dysfunction and Exclusion algorithm to assess key gene expression. For the prognostic model, Spearman correlation analysis was used to explore the relationship between model scores and immunization scores, and the relationship was validated using the R package pheatmap. For cluster analysis, the immune infiltration status under different subgroups was assessed with xCELL, ESTIMATE, IPS, quanTIseq, TIMER, EPIC and CIBERSORT algorithms. Moreover, the association of different subgroups with immune-related molecules, including immunostimulators, chemokines, receptors, immunoinhibitors and MHC molecules, was analyzed.

### Cell lines and culture

Human PCa cell lines (DU145 and LNCaP) were obtained from the surgical laboratory of Zhongda Hospital, Southeast University, and were provided by Dr. Saisai Chen. DU145 and LNCaP were cultured in RPMI 1640 medium (Gibco Thermo Fisher Scientific, USA), containing 10% fetal bovine serum (LONSERA, Uruguay), and 1% penicillin–streptomycin solution (Keygen, China).

### Cell phenotype

The small interfering RNAs (siRNAs) of ERLIN2 and CDK5RAP3 were designed and synthesized by GenePharma Co. (China). For cell proliferation assay, 1000 cells were seeded into 96-well plates for 0–96 h, and 10 µL of the cell counting kit-8 (Keygen, China) solution was added per well. After a 2-h incubation at 37 °C, optical density (OD) at 450 nm was measured on a microplate reader (Bio-Tek, USA). Cells were inoculated onto 6-well plates for the wound-healing assay and treated with si-/nc-ERLIN2 and si-/nc-CDK5RAP3. The cell wound edge was marked and photographed under a microscope at the starting time point, and after 0–24 h, the cells’ migrated distances were measured and analyzed for the wound closure percentage. For migration assays, cells were inoculated into a 24-well transwell cell apical chamber containing matrix gel (BD, USA) for evaluating cell invasion. Cells that invaded the bottom chambers were fixed with 4% polyformaldehyde, stained with 0.1% crystal violet solution, counted, and photographed under a microscope.

### Immunohistochemistry

Immunohistochemical images of key genes in normal individuals and PCa patients were collected with the help of the Human Protein Atlas (HPA) database (https://www.proteinatlas.org).

### Statistical analysis

The statistical analysis of this study was performed automatically by the relevant databases. Differences were statistically significant when *p* < 0.05 or log-rank *p* < 0.05.

## Result

### Identification of ERLIN2 and CDK5RAP3 as ERS-related prognostic genes in PCa

**Fig. 2:**
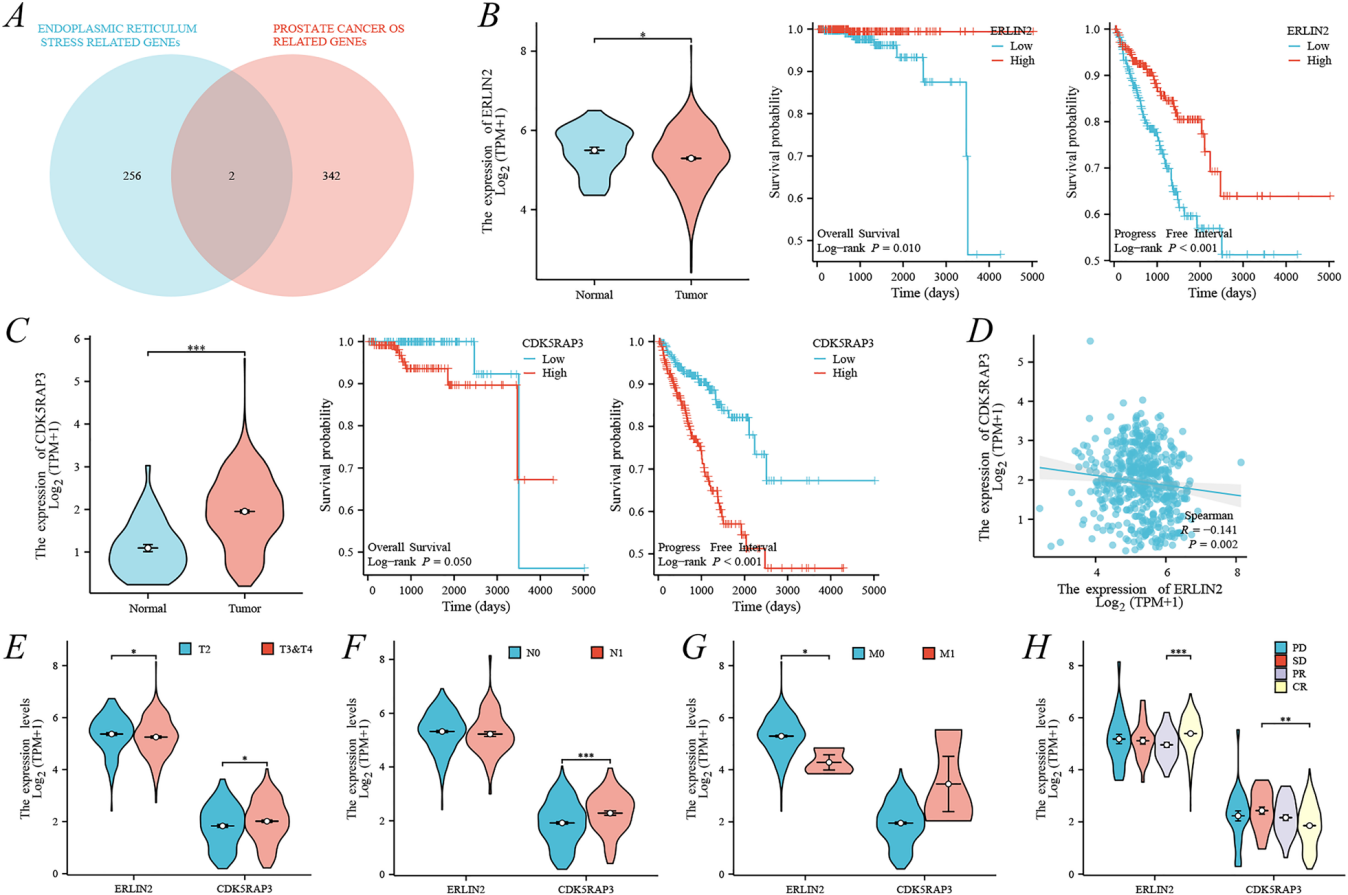
Identification and characterization of prognostic ERS-related genes. **A** Venn diagram of ERS-related genes versus PCa prognostic OS-related genes; in TCGA-PRAD, **B** ERLIN2 differential expression, OS and PFI; **C** CDK5RAP3 differential expression, OS and PFI; **D** correlation of expression between ERLIN2 and CDK5RAP3; correlation between ERLIN2 and CDK5RAP3 with clinic-pathologic features, including **E** T stage, **F** N stage, **G** M stage, and **H** primary treatment outcome

258 ERS-related genes were obtained and intersected with 344 OS-related prognostic genes; ERLIN2 and CDK5RAP3 were ultimately obtained as potentially ERS-related prognostic genes (Fig. 2A). Subsequently, ERLIN2 was verified to be lowly expressed in PCa and associated with a good prognosis in TCGA-PRAD, including OS and PFI (Fig. 2B), whereas CDK5RAP3 was highly expressed in PCa and associated with a bad prognosis (Fig. 2C). Moreover, ERLIN2 and CDK5RAP3 were negatively correlated, *r* = − 0.141, *p* = 0.002 (Fig. 2D). The relationship between basic clinical information and gene expression (ERLIN2 and CDK5RAP3) in the TCGA-PRAD cohort is shown in Additional file 1: Table S1. Association with clinic-pathologic features revealed that ERLIN2 was expressed significantly low in patients with high T stage (T3&4), M stage (M1), and partial remission (PR) (Fig. 2E–H), whereas CDK5RAP3 was expressed significantly high in patients with high T stage (T3&4), N stage (N1), and partial remission (PR) (Fig. 2E–H). Therefore, the present study tentatively suggests that ERLIN2 is a cancer suppressor gene and CDK5RAP3 is an oncogene. Interestingly, ERLIN2 and CDK5RAP3 were correlated with patients’ disease states of complete and partial remission, and this phenomenon implicated that they might influence the outcome of PCa treatment.

**Fig. 3:**
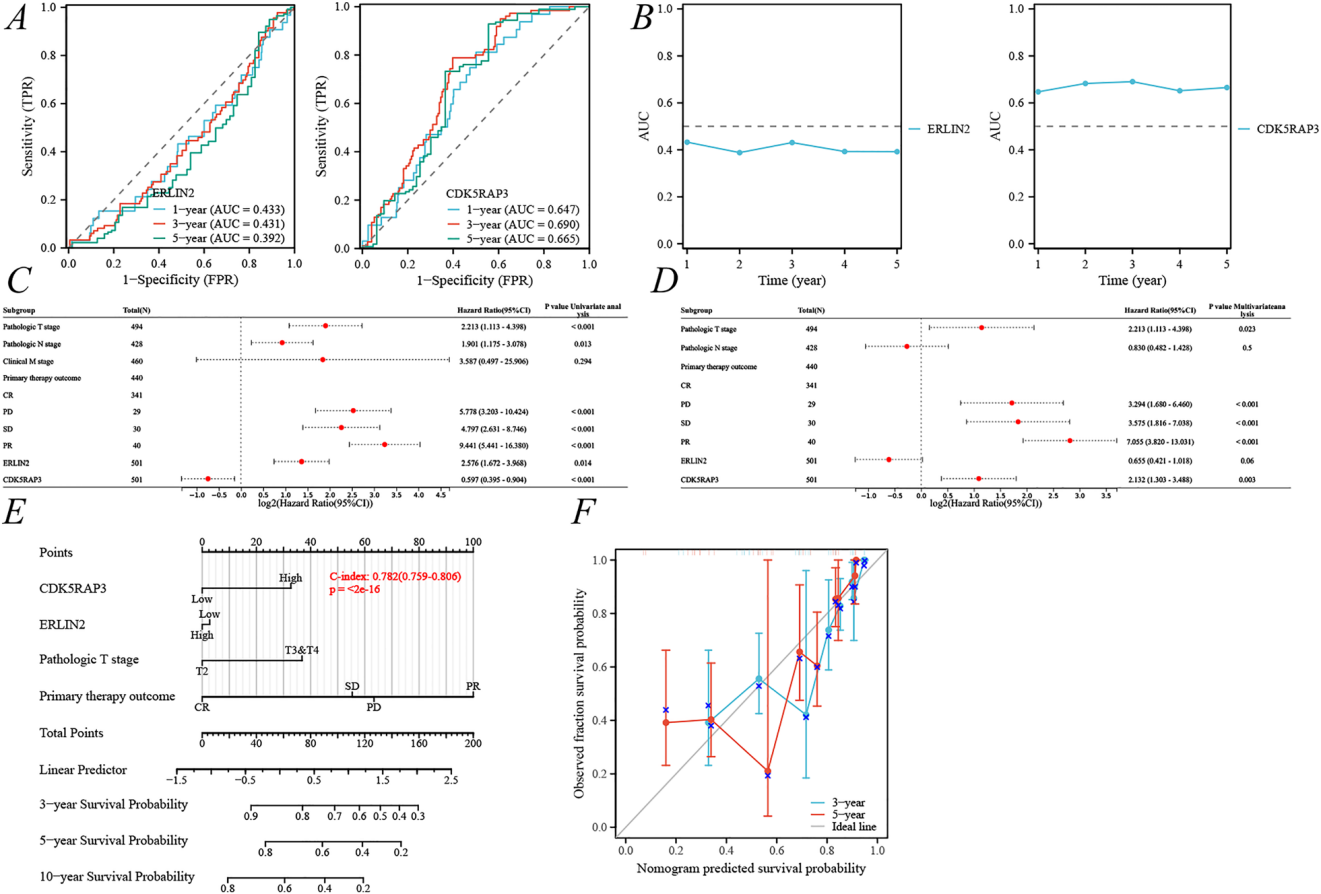
Construction of the PFI nomogram with the clinic-pathological features, ERLIN2 and CDK5RAP3. **A** PFI-associated time-dependent ROC for ERLIN2 and CDK5RAP3; **B** PFI-associated time-dependent AUC for ERLIN2 and CDK5RAP3; prognostic ERS-related genes combined with clinic-pathologic features in univariate (**C**) and multivariate (**D**) Cox regression analyses; **E** PFI nomogram; **F** 3-, 5-year prognostic calibration curves

Due to the specificity of the PCa prognosis, this study first constructed the correlation between time-dependent ROC and univariate–multivariate Cox regression related to OS; However, its results were not statistically significant (Additional file 1: Fig. S1). Therefore, we continued to explore the nomogram with a focus on PFI. The 1-, 3-, and 5-year PFIs for AUC_ERLIN2_ were 0.433, 0.431, and 0.392, respectively (Fig. 3A, B); and the 1-, 3-, and 5-year PFIs for AUC_CDK5RAP3_ were 0.647, 0.690, and 0.665, respectively (Fig. 3A, B). The inclusion of ERLIN2, CDK5RAP3 and clinical characteristics (TNM stage, primary therapy outcome) in the univariate–multivariate Cox regression, ultimately clarified that CDK5RAP3, T stage, and primary therapy outcome had independent prognostic value, but ERLIN2 multivariate Cox regression result of *p* = 0.06, was still included in the construction of the nomogram (Fig. 3C, D). Therefore, this study finally obtained the nomogram based on ERLIN2, CDK5RAP3, T stage, and primary therapy outcome, with C-index = 0.782, *p* < 2e−16 (Fig. 3E); the 3- and 5-year prognostic calibration curve was well fitted to the ideal curve (Fig. 3F).

### Characterization of ERLIN2 and CDK5RAP3 as key genes for immune infiltration and immunotherapy response in PCa

**Fig. 4:**
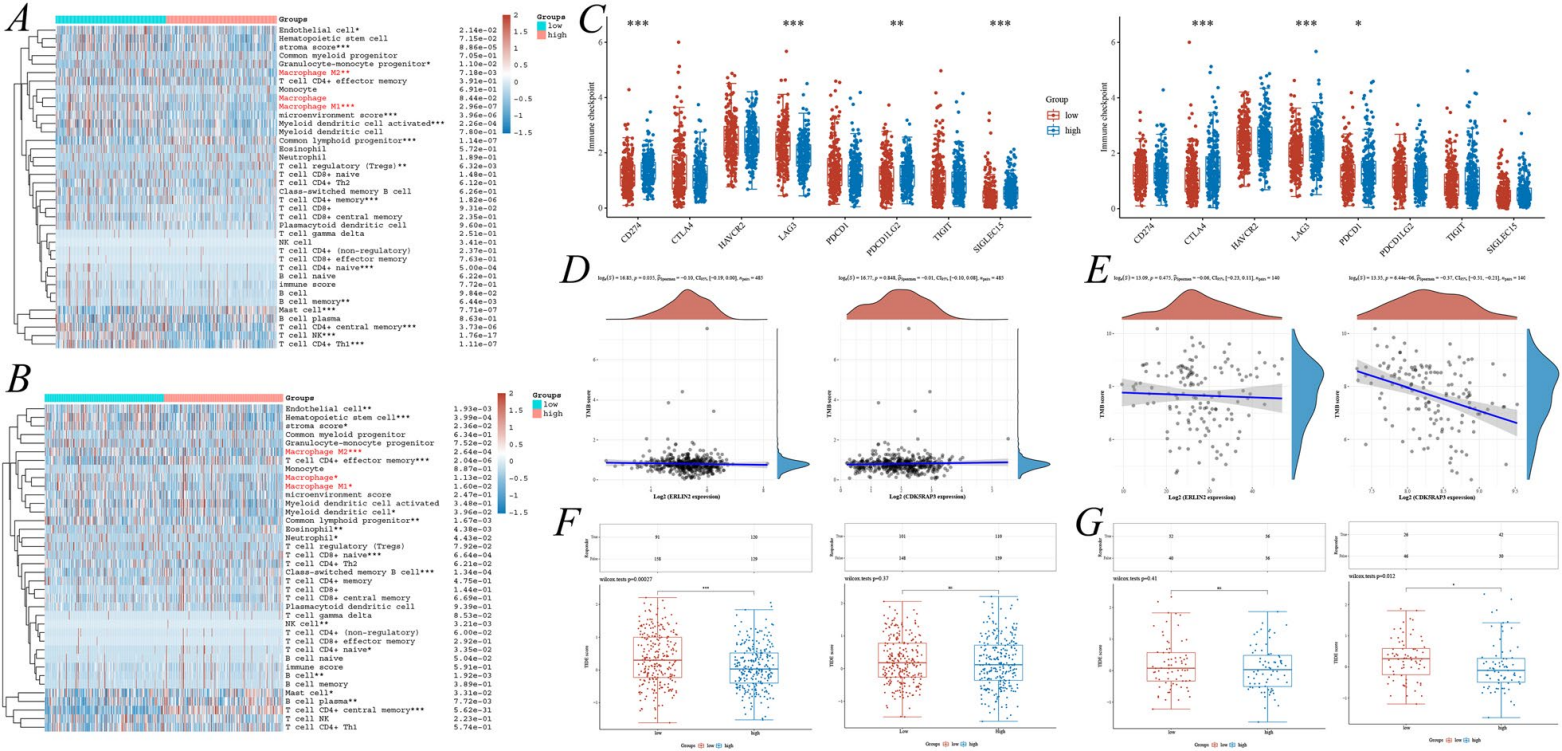
Single gene immunocorrelation analysis of ERLIN2 and CDK5RAP3. Analysis of ERLIN2 (**A**) and CDK5RAP3 (**B**) with immunocyte infiltration in the TCGA-PRAD cohort based on the xCELL algorithm; **C** correlation analysis of ERLIN2 and CDK5RAP3 with immune checkpoint-related genes; correlation analysis of ERLIN2 and CDK5RAP3 with tumor mutation burden (TMB) in TCGA-PRAD (**D**) and ICGA-PRAD (**E**); correlation of ERLIN2 and CDK5RAP3 with immune checkpoint blockade therapy (ICB) in TCGA-PRAD (**F**) and ICGA-PRAD (**G**)

Through the xCELL algorithm, this study found that ERLIN2 and CDK5RAP3 were significantly associated with many tumor-immune cells (Fig. 4A, B). Given the immune microenvironment characterization, patients suffering from PCa having low ERLIN2 expression contained more M1 and M2 macrophages, whereas those with high CDK5RAP3 expression contained fewer M1 and M2 macrophages (Fig. 4A, B). Subsequently, further exploration of ERLIN2, CDK5RAP3, and immunotherapy correlation revealed that ERLIN2 was associated with immune checkpoint-related genes such as CD274, LAG3, PDCD1LG2, and SIGLEC15; whereas CDK5RAP3 was associated with CTLA4, LAG3, and PDCD1 (Fig. 4C). Furthermore, in the TCGA-PARD cohort, ERLIN2 expression was negatively correlated with TMB (*p* = 0.035, *r* = − 0.10) (Fig. 4D), whereas in the ICGA-PRAD cohort, CDK5RAP3 expression was negatively correlated with TMB (*p* = 6.44e−06, *r* = − 0.37) (Fig. 4E). In response to immune checkpoint blockade therapy, responsiveness to low ERLIN2 expression was higher in the TCGA-PARD cohort (Fig. 4F, G); responsiveness to low CDK5RAP3 expression was higher in the ICGA-PRAD cohort (Fig. 4F, G). These results all indicated that ERLIN2 and CDK5RAP3 affected the PCa TIM and had potential relevance to tumor immunotherapy.

### Construction of prognostic models and subgroups based on ERLIN2 and CDK5RAP3 in PCa

**Fig. 5:**
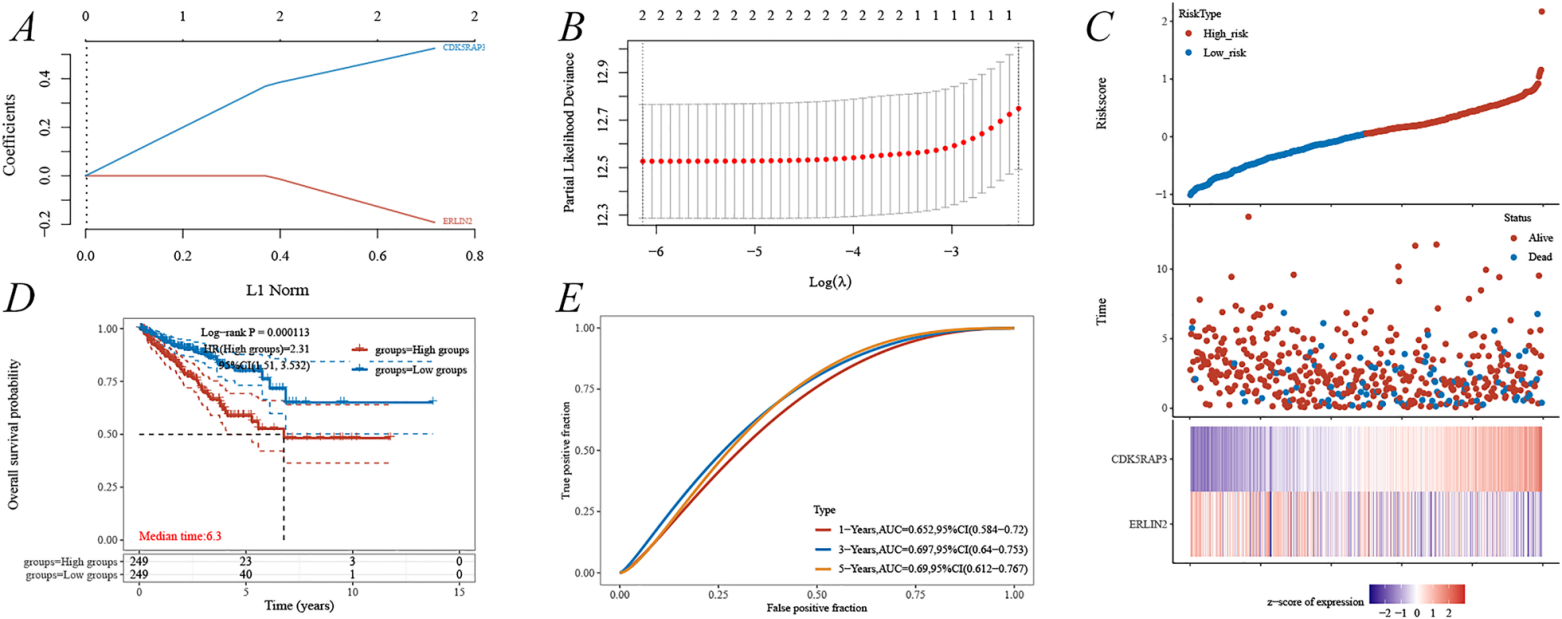
Construction of the PFI-related risk prognostic model about ERLIN2 and CDK5RAP3. **A**, **B** LASSO regression analysis; **C** risk score distribution, survival status, and expression of the two ERS-related genes in TCGA-PRAD; **D** Kaplan–Meier survival curves analyses; **E** time-dependent ROC curves

The prognostic model for PFI based on ERLIN2 and CDK5RAP3 was constructed using LASSO regression analysis with Riskscore = (− 0.1918) * ERLIN2 + (0.5254) * CDK5RAP3, lambda.min = 0.0022 (Fig. 5A, B). Analysis of ERLIN2 and CDK5RAP3 about the survival status of PCa showed that the number of survival statuses was lower in the high-risk group, with low ERLIN2and high CDK5RAP3 expressions (Fig. 5C). The analysis in the TCGA-PRAD cohort suggested a shorter PFI in the high-risk group (Fig. 5D), and the 1-, 3-, and 5-year PFI ROC were 0.652, 0.697, and 0.690, respectively (Fig. 5E). Based on the xCELL algorithm, the prognostic model risk score was analyzed for correlation with immune infiltration, suggesting that the model mainly showed a significant negative correlation with T cell CD4+ central memory and a positive correlation with common lymphoid progenitor (Additional file 1: Fig. S2). Moreover, this study also focused on the positive correlation between risk scores and macrophages (both M1 and M2) in this prognostic model (Additional file 1: Fig. S2).

**Fig. 6:**
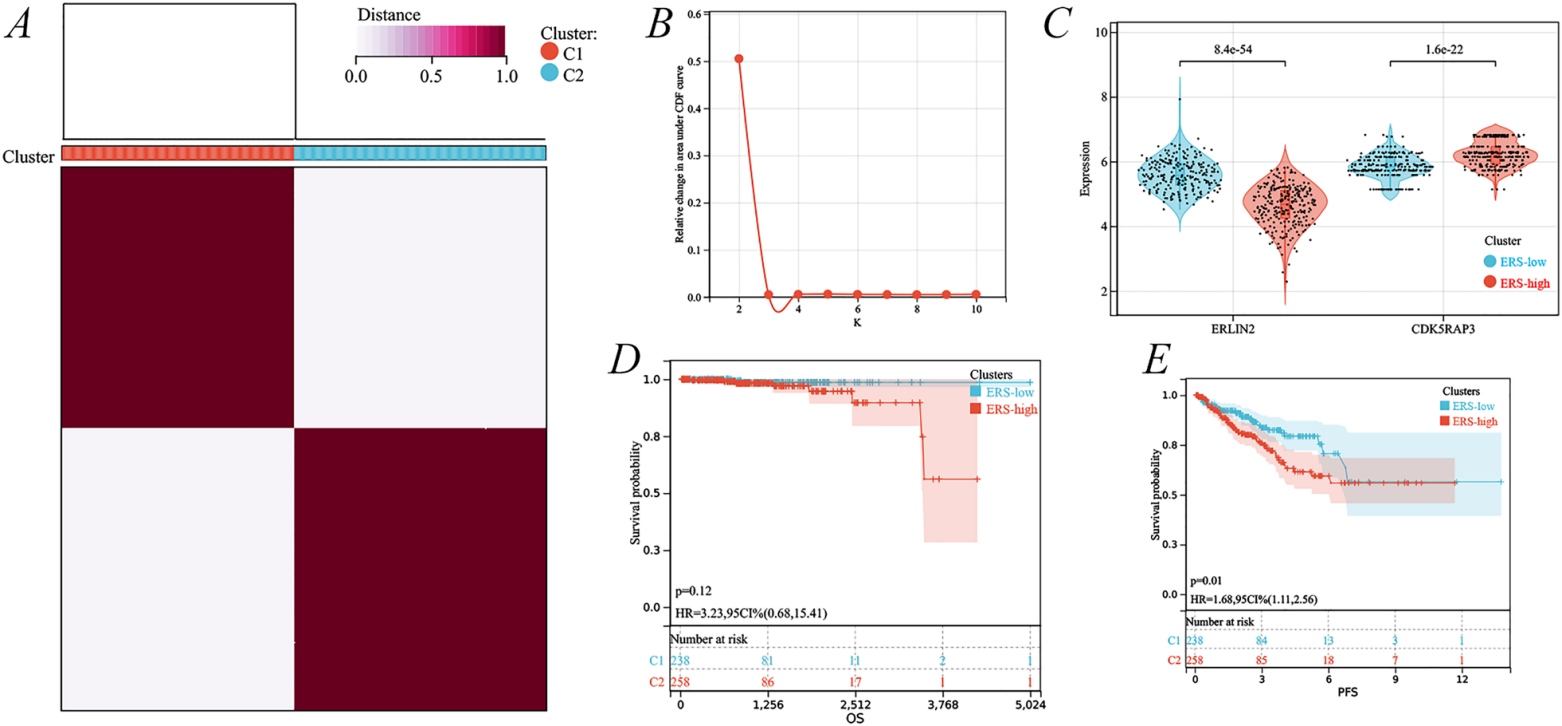
Identification of two cluster subgroups of ERS-related genes. **A** Cluster heatmap at *K* = 2; **B** area under the distribution curve; **C** differences in expression of ERLIN2 and CDK5RAP3 under different cluster subgroups; Kaplan–Meier curves for OS (**D**) and PFI (**E**) for different cluster subgroups

Because only two genes, ERLIN2 and CDK5RAP3, had the highest average concordance within the sample group based on *K* = 2, cluster analysis was performed to obtain two subgroups, with a total of 249 ERS high-risk and 249 ERS low-risk (Fig. 6A, B). Differential expression analysis of different subgroups suggested lower ERLIN2 expression and higher CDK5RAP3 expression in the ERS high-risk group relative to the ERS low-risk group (Fig. 6C). Combined with the results of OS and PFI, there was a decreasing trend in OS in the ERS high-risk group, but it was not statistically significant (Fig. 6D); however, PFI was significantly shorter in the ERS high-risk group (*p* = 0.01) (Fig. 6E).

### Identification of differentially expressed genes and pathways in different ERS-related subgroups

**Fig. 7:**
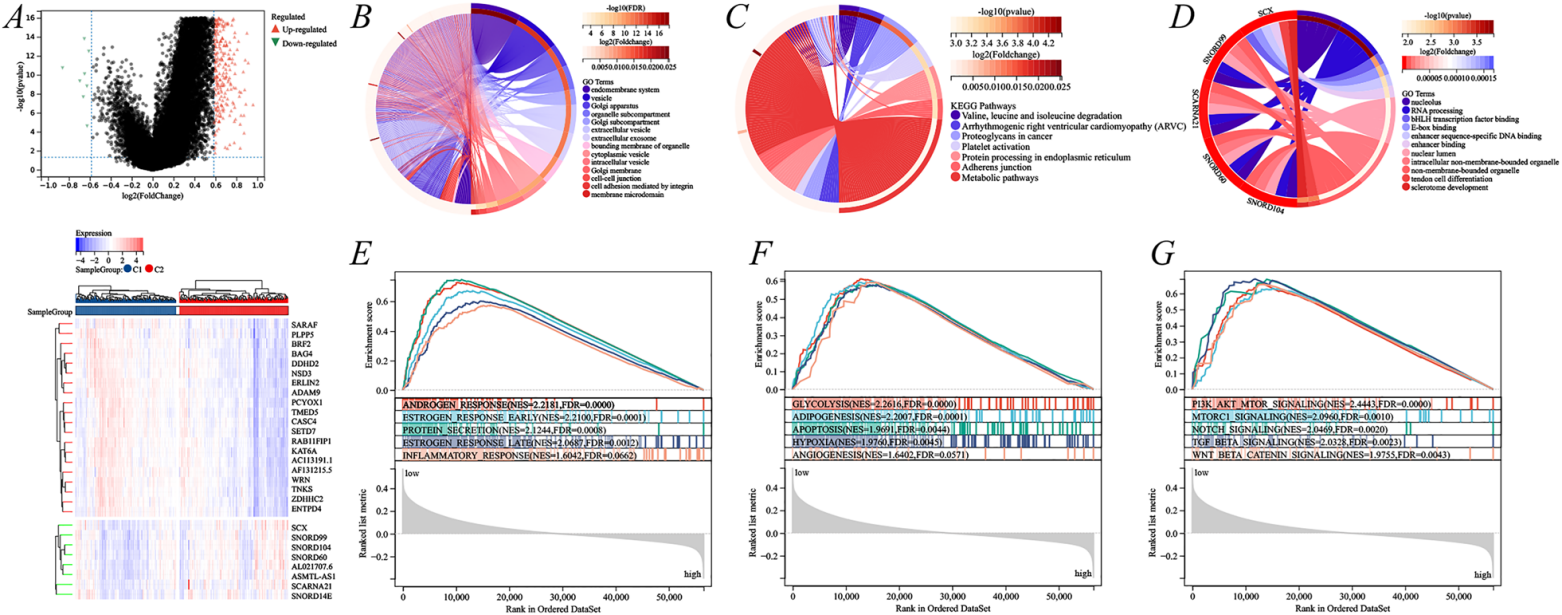
Identification of differentially expressed genes and signaling pathways under different cluster subgroups. **A** Volcano and heat maps of differentially expressed genes (DEGs) profiles; **B** GO analysis of upregulated DEGs; **C** KEGG analysis of upregulated DEGs; **D** GO analysis of downregulated DEGs; **E**–**G** GSEA pathway enrichment analysis of DEGs

To explore the molecular mechanisms underlying the prognostic differences caused by ERS-related subgroups, a total of 526 abnormally regulated genes were identified in the two subgroups, including 518 upregulated and 8 downregulated genes (FC ≥ 1.5, *p* < 0.05) (Fig. 7A). GO analysis revealed that upregulated genes were concentrated in endomembrane system, vesicle, golgi apparatus, and so on. (Fig. 7B); downregulated genes were found in nucleolus, RNA processing, bHLH transcription factor binding, and so on (Fig. 7C). KEGG analysis revealed that upregulated genes were concentrated in valine leucine and isoleucine degradation, arrhythmogenic right ventricular cardiomyopathy, proteoglycans in cancer, and metabolic pathways (Fig. 7D); KEGG analysis could not be performed for downregulated genes due to them being fewer in number. Moreover, Gene Set Enrichment Analysis (GSEA) indicated significant differences between ERS low-risk and ERS high-risk subgroups in multiple intracellular responses (Fig. 7E), metabolic responses (Fig. 7F), and oncogenic signaling pathways (Fig. 7G), which could be potential mechanisms leading to different prognosis. Interestingly, the study discovered that ANDROGEN RESPONSE and ESTROGEN RESPONSE had higher enrichment scores in the ERS high-risk subgroup (Fig. 7E), which may be an important factor influencing androgen deprivation therapy.

Through somatic mutation profiles, this study found significant differences in gene mutations between the ERS low-risk and ERS high-risk subgroups. The first 10 mutated genes in the ERS low-risk subgroup were SPOP, TP53, TTN, FOXA1, MUC16, SPTA1, SYNE1, CACNA1E, CSMD3, and KMT2D (Additional file 1: Fig. S3A); mutated genes in the ERS high-risk subgroup were TP53, TTN, SPOP, FOXA1, KMT2D, LRP1B, KMT2C, SYNE1, MUC16, and RYR1 (Additional file 1: Fig. S3B). Among them, the mutation rates of SPOP and MUC16 were significantly lower in the ERS high-risk than in the ERS low-risk subgroup.

### Exploration of the immune relevance in different ERS-related gene subgroups

**Fig. 8:**
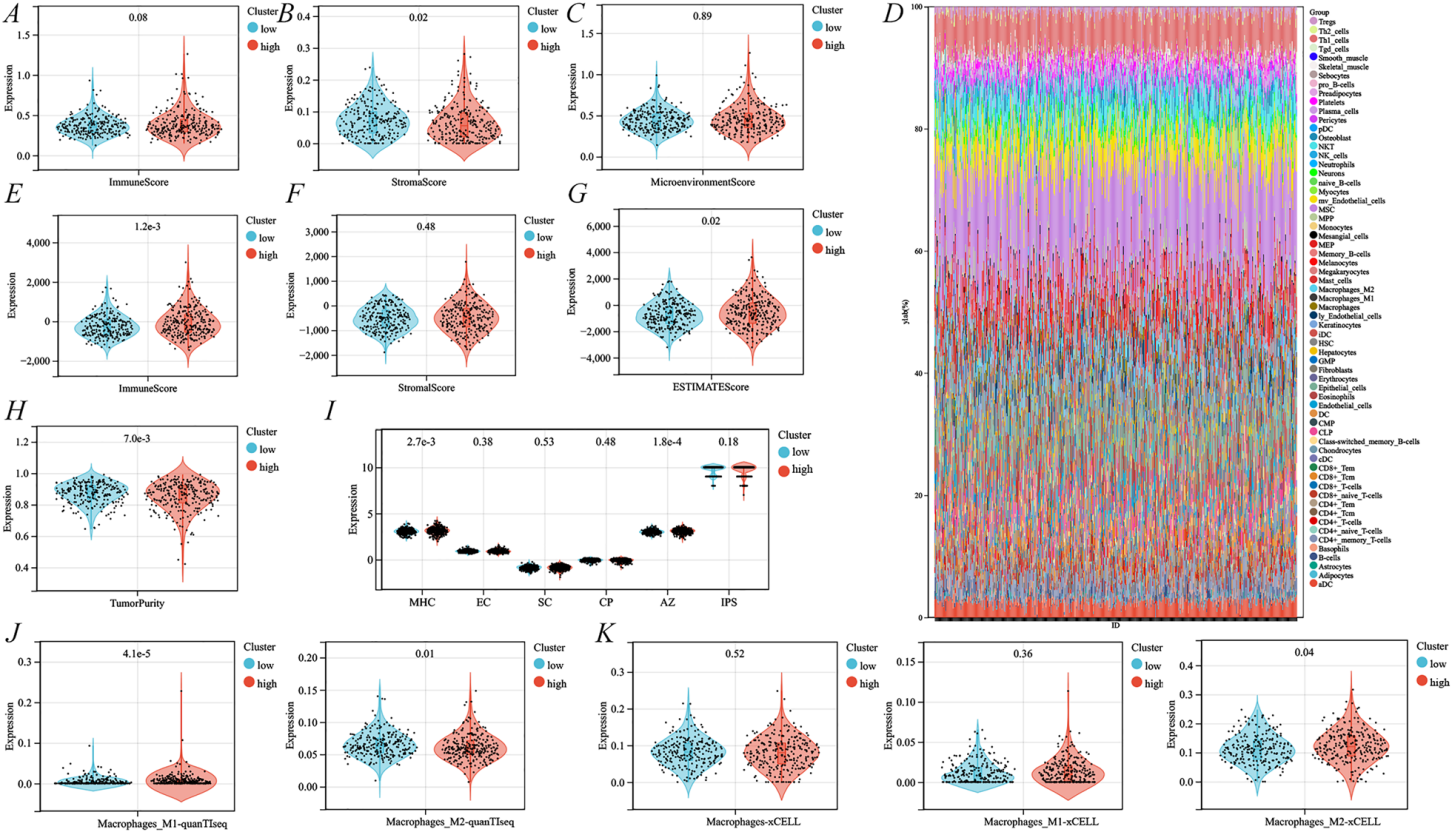
Recognition of immune infiltration differences under different cluster subgroups. ImmueScore (**A**), StromalScore (**B**) and MicroenvironmentScore (**C**) based on the xCELL algorithm; **D** immunocyte infiltration stack plot for different cluster subgroups under the xCELL algorithm; ImmueScore (**E**), StromalScore (**F**), ESTIMATE score (**G**) and tumorpurity (**H**) based on the ESTIMATE algorithm; **I** IPS scores for different cluster subgroups; differential expression of M1 and M2 macrophages under quanTIseq (**J**) and xCELL algorithm (**K**) with different cluster subgroups

As the above studies initially confirmed the correlation of ERLIN2 and CDK5RAP3 with the PCa-immune microenvironment and immunotherapy, the study delved into the correlation between prognostic subgroups based on ERLIN2-CDK5RAP3 with tumor immunity. By the xCELL and ESTIMATE algorithms, the ERS high-risk subgroup was identified to have higher ImmueScore, StromalScore, and ESTIMATE Score, and lower Tumor purity (Fig. 8A–C, E–H), and immune cell infiltration was explored under different subgroups according to the xCELL algorithm (Fig. 8D). IPS scores suggested higher MHC and AZ expression in ERS high-risk subgroup (Fig. 8I). Furthermore, as existing studies confirmed that both PCa and ERS are significantly correlated with macrophages, this study used the quantTIseq, xCELL, and CIBERSORT algorithms containing M1 and M2 macrophage subtypes, as well as discovered that quantTIseq suggested that the ERS high-risk subgroup had more M1 macrophages and fewer M2 macrophages (Fig. 8J), whereas xCELL suggested that the ERS high-risk subgroup had more both M1 and M2 macrophages (Fig. 8K), and there was no statistical significance for the CIBERSORT analysis (Additional file 1: Fig. S4C). Moreover, different ERS-related subgroups were confirmed to be significantly associated with macrophage by TIMER and EPIC algorithms (Additional file 1: Fig. S4A, B). Confusingly, there is not yet a clear homogeneity in macrophage expression for prognostic subgroups under different algorithms, which may be due to differences caused by different algorithmic folding or macrophage identification. Therefore, the correlation between ERS-related gene subgroups and macrophage expression needs to be further confirmed by experimental studies.

**Fig. 9:**
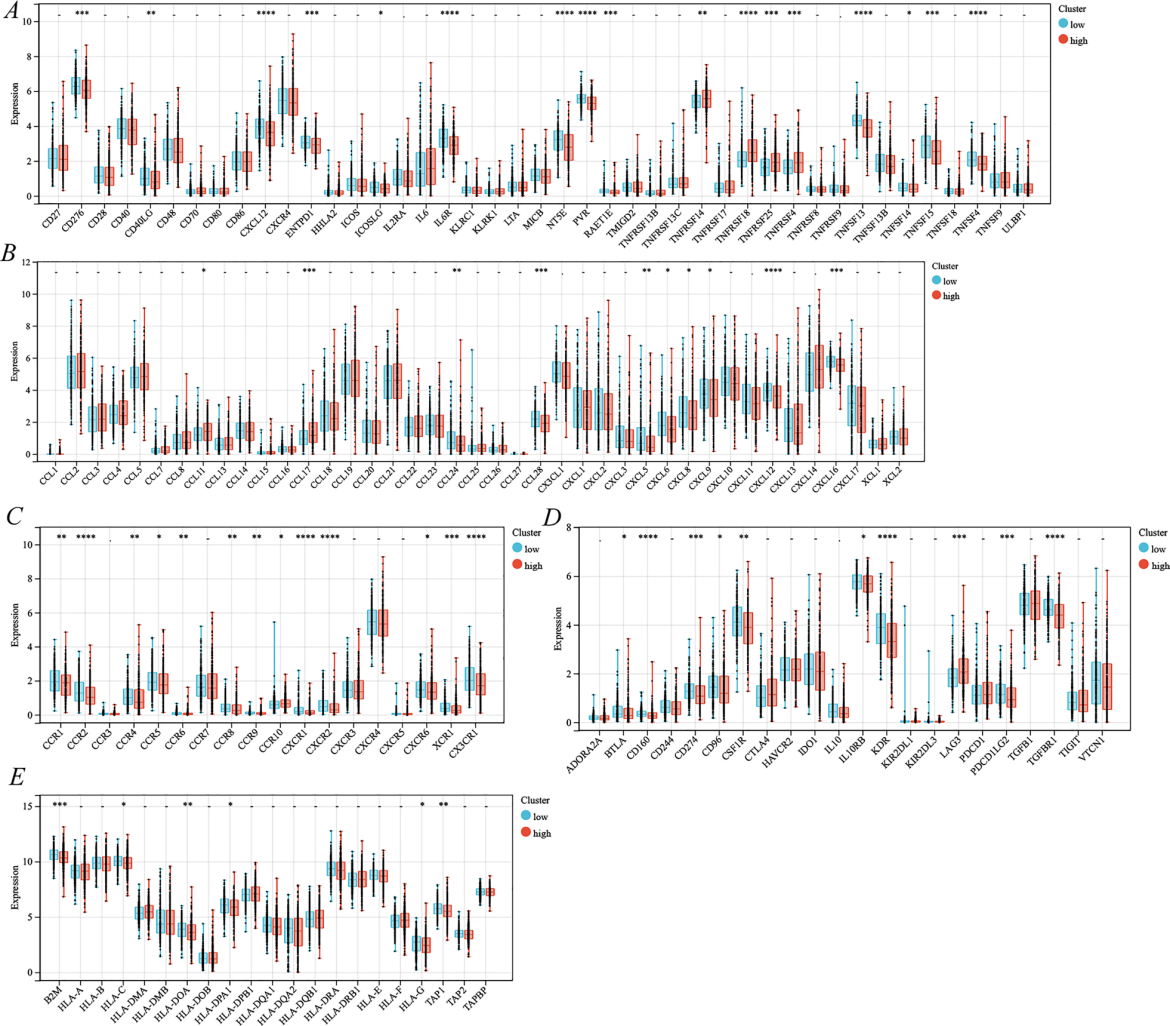
Identification of immune-related molecules expression under different cluster subgroups. **A** Immunostimulators; **B** chemokines; **C** receptors; **D** immunoinhibitors; **E** MHC molecules

Subsequently, this study thoroughly explored the correlation of different subgroups with immune molecules, including immunostimulators, chemokines, receptors, immunoinhibitors, and MHC molecules. Multiple immunostimulators were expressed significantly low in the ERS high-risk subgroup (*p* < 0.001), including CD276, CXCL12, ENTPD1, IL6R, NT5E, PVR, RAET1E, TNFRSF18, TNFRSF25, TNFRSF4, TNFSF13, TNFSF15, and TNFSF4 (Fig. 9A). Additionally, the immunoinhibitor LAG3 was significantly highly expressed in the ERS high-risk subgroup (*p* < 0.001), but CD160, CD274, KDR, PDCD1LG2, and TGFBR1 were expressed significantly less (Fig. 9D). The chemokine CCL17 was significantly highly expressed in the ERS high-risk subgroup, whereas CCL28, CXCL12, and CXCL16 were expressed significantly less (Fig. 9B). The ERS high-risk subgroup showed significantly low expression of the receptors CCR2, CXCR1, CXCR2, XCR1, and CX3CR1 (Fig. 9C). Finally, for MHC molecules, only B2M showed significantly low expression in the ERS high-risk subgroup (Fig. 9E).

### Verification of ERLIN2 and CDK5RAP3 expression in PCa by cell phenotype and immunohistochemistry

**Fig. 10:**
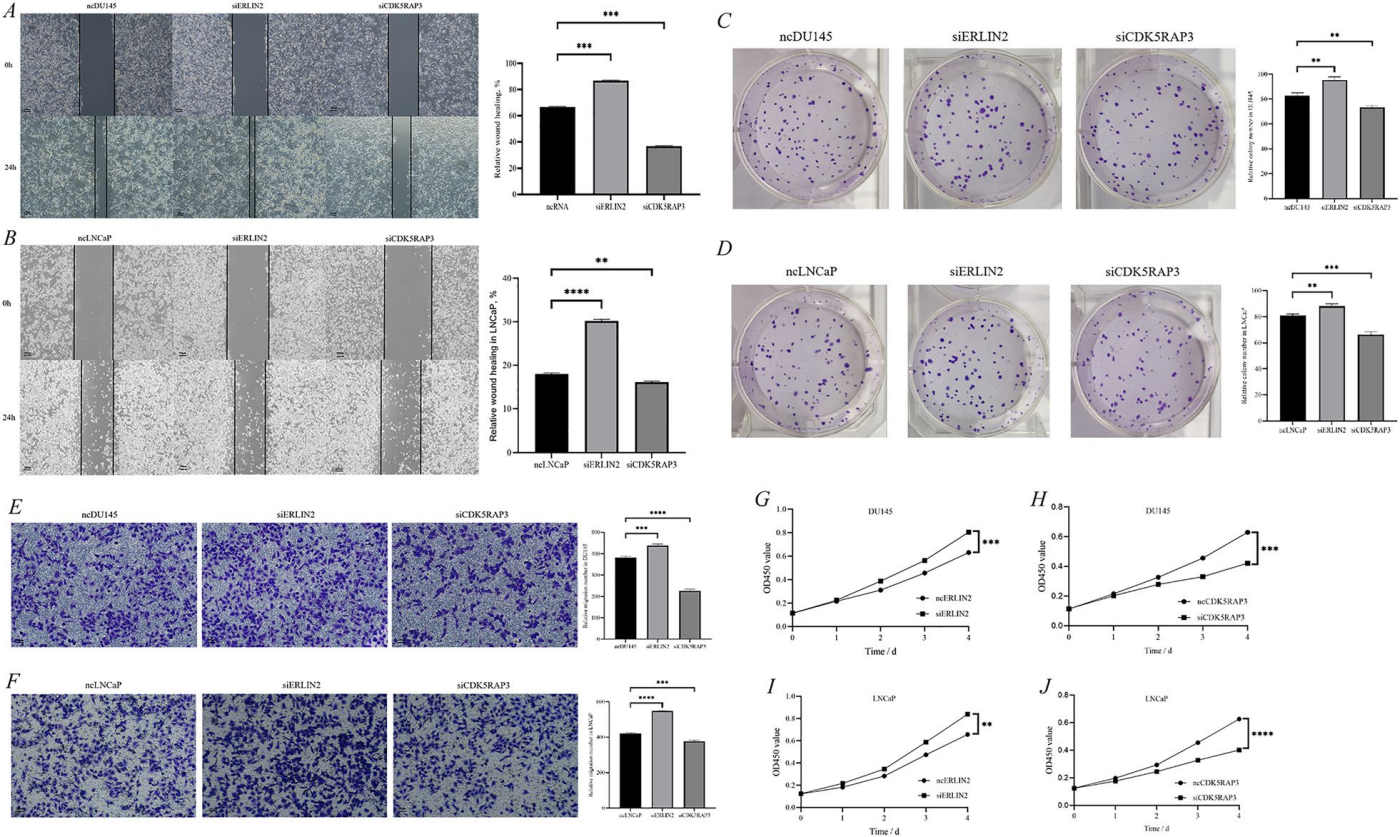
ERLIN2 and CDK5RAP3 were proved to promote prostate cancer progression in vivo. Scratch tests in DU145 (**A**) and LNCaP (**B**); clone formation assay in DU145 (**C**) and LNCaP (**D**); migration assay in DU145 (**E**) and LNCaP (**F**); ERLIN2 (**G**) and CDK5RAP3 (**H**), CCK8 assay in DU145; ERLIN2 (**I**) and CDK5RAP3 (**J**) CCK8 assay in LNCaP; left, wound healing and migration assay, scale bar, 100 μm

The relative wound healing rate of cells was found to be significantly higher after siERLIN2 and significantly lower after siCDK5RAP3 by cell scratch assay (Fig. 10A, B). Then, CCK8 experiments suggested accelerated cell proliferation after siERLIN2, whereas cell proliferation was significantly weakened after siCDK5RAP3 (Fig. 10G–J). In addition, the number of cell clones increased after siERLIN2, while the number of cell clones decreased after siCDK5RAP3, compared with the ncRNA group (Fig. 10C, D). Moreover, the cell migration ability was elevated after siERLIN2, whereas it was decreased after siERLIN2 (Fig. 10E, F). Collectively, the above studies further confirmed ERLIN2 as an anti-oncogene and CDK5RAP3 as a pro-oncogene in PCa.

**Fig. 11:**
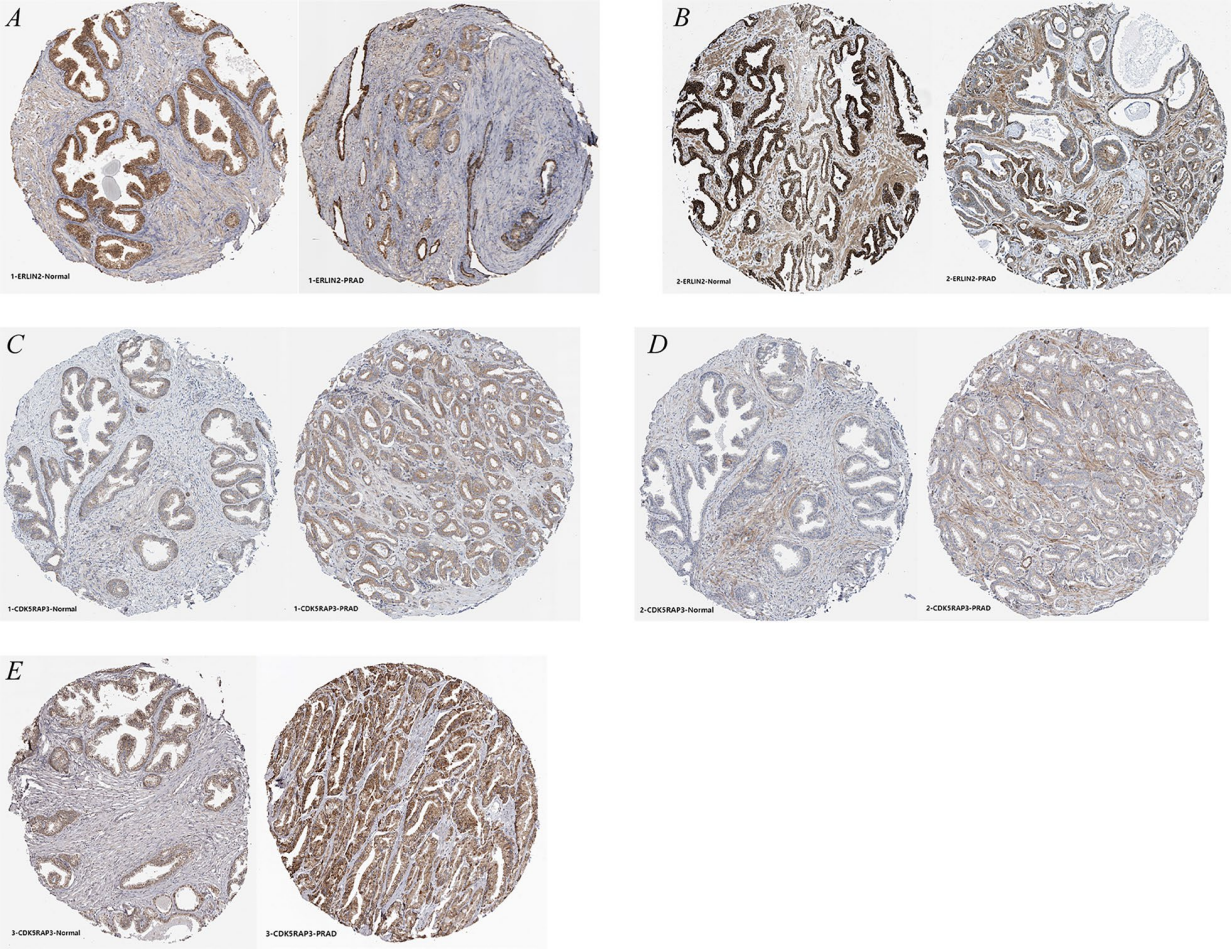
Immunohistochemistry of ERLIN2 and CDK5RAP3 in the HPA database. **A**, **B** ERLIN2; **C**–**E** CDK5RAP3

Through the HPA database, immunohistochemical images and patient information under different antibody identifications were obtained (Additional file 1: Table S2). Antibodies HPA002025 (Fig. 11A) and CAB014894 (Fig. 11B) directed against ERLIN2 suggested that ERLIN2 had low expression in PCa tissues; antibodies HPA022141 (Fig. 11C), HPA022882 (Fig. 11D), and HPA027883 (Fig. 11E) directed against CDK5RAP3 suggested that CDK5RAP3 was highly expressed in PCa tissues. The above results were consistent with the results of the previous bioinformatic analyses.

## Discussion

Prostate cancer has become the third most common cancer, accounting for about 7% of cancer-related deaths in men worldwide [1]. The early-stage patients have a good prognosis [11], while the late-stage patients have a high mortality rate due to metastasis, invasion and drug resistance [12]. Due to the resistance to denervation and chemotherapy in PCa, and unsatisfactory therapeutic effects of immune monotherapy [2–6], studies are more focused on the exploration of its TIM and the identification of more effective immunotherapeutic targets. Endoplasmic reticulum stress, as an important protective stress response, is closely related to the TIM, especially to the inflammatory response regulated by macrophage polarization [8, 9]. Moreover, PCa, as a cold tumor, has an immune characterized by lymphocyte deficiency and macrophage infiltration [7], which suggests that ERS may be a potentially important mechanism for the formation of the immune microenvironment in PCa. Currently, there are no comprehensive bioinformatics analyses exploring the overall role of ERS-related genes, and the critical role of ERS affecting macrophage polarization in tumor progression and drug resistance has not been clarified in PCa.

This study eventually identified two key genes with important prognostic and immunologic value among 258 ERS-related genes, including ERLIN2 and CDK5RAP3. In particular, the above genes have not been recognized and confirmed in PCa, and they are of great research value. Endoplasmic reticulum lipid raft-associated protein 2 (ERLIN2), a protein containing a conserved SPFH structural domain within the ER membrane, degrades the ER by binding to ER-associated degradation substrates, such as activated inositol trisphosphate receptors [13, 14]. Moreover, ERLIN2 activates sterol regulatory element-binding protein 1c, which maintains high intracellular lipid expression, allowing tumorigenesis to gain a growth advantage [15], including breast cancer [16] and lung adenocarcinomas [17]. No relevant studies have explored the relationship between ERLIN2 and macrophages. Cyclin-dependent kinase 5 regulatory subunit-associated protein 3 (CDK5RAP3) was originally identified as a binding protein for the CDK5 activator p35, which is involved in various cellular processes such as the cell cycle, ERS, UFMylation modification, cell invasion, signaling, autophagy, and apoptosis [18]. CDK5RAP3 plays an essential role in maintaining ER homeostasis through UFMylation couples [18, 19]; Additionally, CDK5RAP3 binds to DDRGK1, an ER membrane protein, to regulate ER-associated proteins [20]. Professor Chang-Ming Huang and colleagues confirmed that in gastric cancer, CDK5RAP3 inhibited nuclear transcription of NF-κB, thereby decreasing the secretion of cytokines IL4 and IL10 and blocking the polarization of M2 macrophages [21]. However, the pro- and anti-cancer roles of CDK5RAP3 are unclear. It plays a tumor suppressor-related role in gastric and renal cancers, but has a pro-carcinogenic role in lung, breast, and cervical cancers [18]. In this study, it was initially clarified that ERLIN2 is an anti-cancer factor and CDK5RAP3 is a pro-cancer factor in PCa, and that the expressions of both were significantly correlated with the clinic-pathologic features.

This study clarified that CDK5RAP3, T stage, and primary treatment outcome were independent prognostic factors for PCa; as well as ERLIN2 and CDK5RAP3 were significantly correlated with immune infiltration (especially macrophages) and immune response. Nomogram and prognostic models were constructed in the TCGA-PRAD cohort with Riskscore = (− 0.1918) * ERLIN2 + (0.5254) * CDK5RAP3, whose above results were beneficial in assisting the prediction of clinical prognostic status of PCa patients. Based on ERLIN2 and CDK5RAP3, cluster analysis was performed, and ERS high-risk and ERS low-risk subgroups were obtained. There were a total of 526 regulated abnormal genes between different subgroups that regulated multiple signaling pathways. Interestingly, ANDROGEN RESPONSE and ESTROGEN RESPONSE were detected to have higher enrichment scores in ERS high-risk subgroups, implying that the above subgroups may influence the choice of androgen deprivation therapy. Recent studies revealed that androgens and thyroid hormone metabolism were closely linked, and that their interactions could mediate PCa formation and progression [22]. Moreover, we classified the high-risk subgroup as a pro-cancer group with lower mutation rates of key genes (SPOP and MUC16), multiple low expression of immune-associated molecules, and differential expression of macrophages (M1 and M2). Related studies suggested that genetic mutations within PCa were associated with disease aggressiveness and poor prognosis, such as BRCA germline mutations [23]. The differential expressions of ERLIN2 and CDK5RAP3 were also validated in normal prostate tissue and PCa. Finally, ERLIN2 as an anti-oncogene and CDK5RAP3 as a pro-oncogene were further confirmed by cell phenotype assays and immunohistochemistry. The above results initially clarified that ERLIN2 and CDK5RAP3 were important ERS-related genes in PCa, which had high prognostic and immunological values, and could support patients’ clinical prognosis and treatment selection based on prognostic models and cluster subgroups.

However, this study also contained some limitations. In this paper, ERLIN2 and CDK5RAP3 were initially identified as potential biomarkers by bioinformatics, and were not further validated in clinical patients. Currently, due to the widespread use of prostate-specific antibodies leading to overdiagnosis rates, the Prostate Health Index (PHI), a composite index combining tPSA, fPSA, and p2PSA, was proven to be effective in the assessment of clinical features as well as the prognostic status in PCa, including in combination with the Proclarix or multiparametric magnetic resonance indices [25, 26]. The follow-up of this study hoped to deepen the combination of PHI and other clinical indicators, and to improve the clinical application value of ERLIN2 and CDK5RAP3 through translational medicine.

## Conclusion

In this study, we clarified that ERLIN2 and CDK5RAP3 were ERS-related genes with important prognostic and immunological values in PCa by comprehensive bioinformatics analysis and simple basic experiment, and established the clinic-pathologically relevant prognostic nomogram, as well as the risk prognostic model Riskscore = (− 0.1918) * ERLIN2 + (0.5254) * CDK5RAP3. Cluster analysis identified a pro-cancer role for the ERS high-risk subgroup, which had lower mutation rates of key genes (SPOP and MUC16), multiple low-expressed immune-related molecules, and differential expression in macrophages (M1 and M2). Finally, ERLIN2 as an anti-oncogene and CDK5RAP3 as a pro-oncogene were further confirmed by cell phenotype assays and immunohistochemistry. This study contributed to an intensive exploration of ERS in PCa, finding new prognostic markers, immunotherapeutic targets, and prognosis assessment bases to effectively improve the disease outcome and prevention of PCa.

### Supplementary Information

**Additional file 1: Figure S1.** Overall survival with combined clinic-pathologic characteristics of ERLIN2 and CDK5RAP3. OS related time-dependent ROC for ERLIN2 (A) and CDK5RAP3 (B); Univariate (C) and multivariate (D) Cox regression analyses for prognostic genes combined with clinicopathologic characteristics in PCa. **Figure S2.** Correlation of risk scores with immunocyte infiltration based on the xCELL algorithm for the PFI prognostic model. **Figure S3.** Identification of the top 10 mutated genes under different cluster subgroups. Mutations under low-risk (A) and high-risk (B) subgroups. **Figure S4.** Identification of macrophage expression differences under different cluster subgroups based on multiple algorithms. (A) TIMER; (B) EPIC; (C) CIBERSORT. **Table S1.** The information table of clinic-pathological features associated with the genes in TCGA-PRAD. **Table S2.** Basic information on immunohistochemistry patients in the HPA database.

## Data Availability

The datasets generated and/or analyzed during the current study are available in the Cancer Genome Atlas (TCGA) repository, [https://portal.gdc.cancer.gov/].

## References

[1] Sung H (2021). Global cancer statistics 2020: GLOBOCAN estimates of incidence and mortality worldwide for 36 cancers in 185 countries. CA Cancer J Clin.

[2] Beer TM (2014). Enzalutamide in metastatic prostate cancer before chemotherapy. N Engl J Med.

[3] Harris WP, Mostaghel EA, Nelson PS, Montgomery B (2009). Androgen deprivation therapy: progress in understanding mechanisms of resistance and optimizing androgen depletion. Nat Clin Pract Urol.

[4] Petrylak DP (2004). Docetaxel and estramustine compared with mitoxantrone and prednisone for advanced refractory prostate cancer. N Engl J Med.

[5] Maselli FM (2023). Immunotherapy in prostate cancer: state of art and new therapeutic perspectives. Curr Oncol.

[6] Rebuzzi SE (2022). Immune checkpoint inhibitors in advanced prostate cancer: current data and future perspectives. Cancers.

[7] Shimura S (2000). Reduced infiltration of tumor-associated macrophages in human prostate cancer: association with cancer progression. Cancer Res.

[8] Diaz-Bulnes P, Saiz ML, Lopez-Larrea C, Rodriguez RM (2019). Crosstalk between hypoxia and ER stress response: a key regulator of macrophage polarization. Front Immunol.

[9] Sukhorukov VN (2020). Endoplasmic reticulum stress in macrophages: the vicious circle of lipid accumulation and pro-inflammatory response. Biomedicines.

[10] Storm M, Sheng X, Arnoldussen YJ, Saatcioglu F (2016). Prostate cancer and the unfolded protein response. Oncotarget.

[11] Trewartha D, Carter K (2013). Advances in prostate cancer treatment. Nat Rev Drug Discov.

[12] Zong Y, Goldstein AS (2013). Adaptation or selection–mechanisms of castration-resistant prostate cancer. Nat Rev Urol.

[13] Zhang X (2015). A novel ER-microtubule-binding protein, ERLIN2, stabilizes Cyclin B1 and regulates cell cycle progression. Cell Discov.

[14] Pearce MM, Wormer DB, Wilkens S, Wojcikiewicz RJ (2009). An endoplasmic reticulum (ER) membrane complex composed of SPFH1 and SPFH2 mediates the ER-associated degradation of inositol 1,4,5-trisphosphate receptors. J Biol Chem.

[15] Wang G (2012). Endoplasmic reticulum factor ERLIN2 regulates cytosolic lipid content in cancer cells. Biochem J.

[16] Wang G (2012). ERLIN2 promotes breast cancer cell survival by modulating endoplasmic reticulum stress pathways. BMC Cancer.

[17] Liu Y (2020). Molecular and immune characteristics for lung adenocarcinoma patients with ERLIN2 overexpression. Front Immunol.

[18] Sheng L, Li J, Rao S, Yang Z, Huang Y (2021). Cyclin-dependent kinase 5 regulatory subunit associated protein 3: potential functions and implications for development and disease. Front Oncol.

[19] Gerakis Y, Quintero M, Li H, Hetz C (2019). The UFMylation system in proteostasis and beyond. Trends Cell Biol.

[20] Wu J, Lei G, Mei M, Tang Y, Li H (2010). A novel C53/LZAP-interacting protein regulates stability of C53/LZAP and DDRGK domain-containing protein 1 (DDRGK1) and modulates NF-kappaB signaling. J Biol Chem.

[21] Wang JB (2023). CDK5RAP3 acts as a tumour suppressor in gastric cancer through the infiltration and polarization of tumour-associated macrophages. Cancer Gene Ther.

[22] Miro C, Di Giovanni A, Murolo M, Cicatiello AG, Nappi A, Sagliocchi S, Di Cicco E, Morra F, Celetti A, Pacifico F, Imbimbo C, Crocetto F, Dentice M (2022). Thyroid hormone and androgen signals mutually interplay and enhance inflammation and tumorigenic activation of tumor microenvironment in prostate cancer. Cancer Lett.

[23] Crocetto F, Barone B, Caputo VF, Fontana M, de Cobelli O, Ferro M (2021). BRCA germline mutations in prostate cancer: the future is tailored. Diagnostics.

[24] Liotti A, La Civita E, Cennamo M, Crocetto F, Ferro M, Guadagno E, Insabato L, Imbimbo C, Palmieri A, Mirone V, Liguoro P, Formisano P, Beguinot F, Terracciano D (2021). Periprostatic adipose tissue promotes prostate cancer resistance to docetaxel by paracrine IGF-1 upregulation of TUBB2B beta-tubulin isoform. Prostate.

[25] Gentile F, La Civita E, Della Ventura B, Ferro M, Cennamo M, Bruzzese D, Crocetto F, Velotta R, Terracciano D (2022). A combinatorial neural network analysis reveals a synergistic behaviour of multiparametric magnetic resonance and prostate health index in the identification of clinically significant prostate cancer. Clin Genitourin Cancer.

[26] Terracciano D, La Civita E, Athanasiou A, Liotti A, Fiorenza M, Cennamo M, Crocetto F, Tennstedt P, Schiess R, Haese A, Ferro M, Steuber T (2022). New strategy for the identification of prostate cancer: the combination of Proclarix and the prostate health index. Prostate.

