# Correction: Endoplasmic reticulum stress-related genes as prognostic and immunogenic biomarkers in prostate cancer

**DOI:** 10.1186/s40001-024-01865-w

**Published:** 2024-06-02

**Authors:** Lilin Wan, Yunxia Fan, Tiange Wu, Yifan Liu, Ruixin Zhang, Saisai Chen, Chenggui Zhao, Yifeng Xue

**Affiliations:** 1 Southeast University, 87 Dingjia Bridge Hunan Road, Nanjing, 210009 China; 2 Department of Urology, Zhongda Hospital Southeast University, 87 Dingjia Bridge Hunan Road, Nanjing, 210009 China; 3 Department of Laboratory, Zhongda Hospital Southeast University, 87 Dingjia Bridge Hunan Road, Nanjing, 210009 China; 4 Department of Urology, Jintan Afliated Hospital of Jiangsu University, No. 500, Jintan Avenue, Jintan District, Changzhou, 213200 China

**Correction: European Journal of Medical Research (2024) 29:242** 10.1186/s40001-024-01818-3

In this article the statement in the Funding information section was incorrectly given as “Changzhou Health Commission Project (ZD2020034)” and should have read “Changzhou Health Commission Project (ZD202034)".

The original article has been corrected.

