# Supplementary material for: Endoplasmic reticulum stress-related genes as prognostic and immunogenic biomarkers in prostate cancer
Source: Eur J Med Res. 2024 Apr 20;29:242. doi: 10.1186/s40001-024-01818-3 (PMC11031923; doi:10.1186/s40001-024-01818-3)
Supplement: Supplementary file 1 — Additional file 1: Figure S1. Overall survival with combined clinic-pathologic characteristics of ERLIN2 and CDK5RAP3. OS related time-dependent ROC for ERLIN2 (A) and CDK5RAP3 (B); Univariate (C) and multivariate (D) Cox regression analyses for prognostic genes combined with clinicopathologic characteristics in PCa. Figure S2. Correlation of risk scores with immunocyte infiltration based on the xCELL algorithm for the PFI prognostic model. Figure S3. Identification of the top 10 mutated genes under different cluster subgroups. Mutations under low-risk (A) and high-risk (B) subgroups. Figure S4. Identification of macrophage expression differences under different cluster subgroups based on multiple algorithms. (A) TIMER; (B) EPIC; (C) CIBERSORT. Table S1. The information table of clinic-pathological features associated with the genes in TCGA-PRAD. Table S2. Basic information on immunohistochemistry patients in the HPA database. [file 40001_2024_1818_MOESM1_ESM.docx]

**Additional file 1**

**
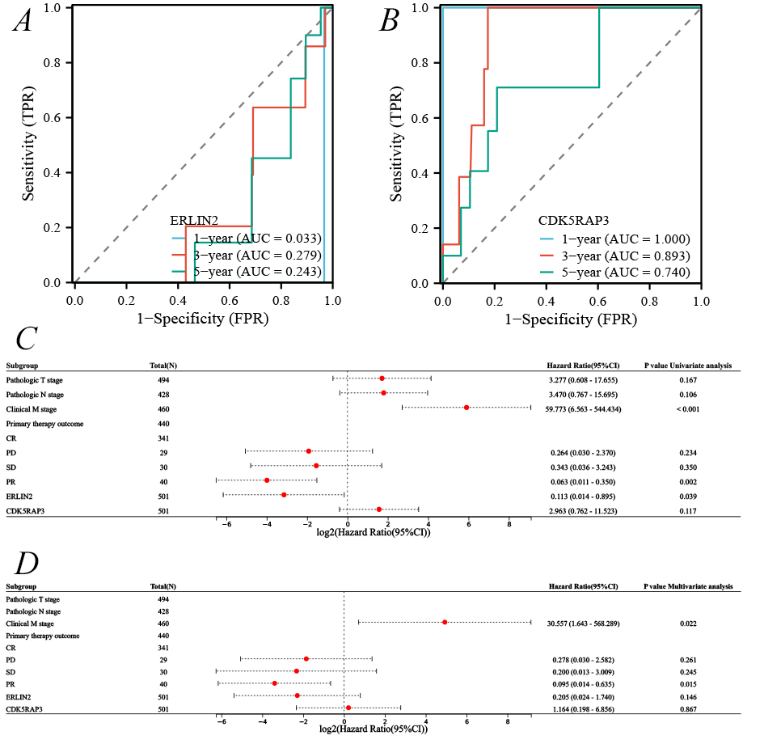
**

**Figure S1: Overall survival with combined clinic-pathologic characteristics of ERLIN2 and CDK5RAP3.** OS related time-dependent ROC for ERLIN2 (A) and CDK5RAP3 (B); Univariate (C) and multivariate (D) Cox regression analyses for prognostic genes combined with clinicopathologic characteristics in PCa.

**
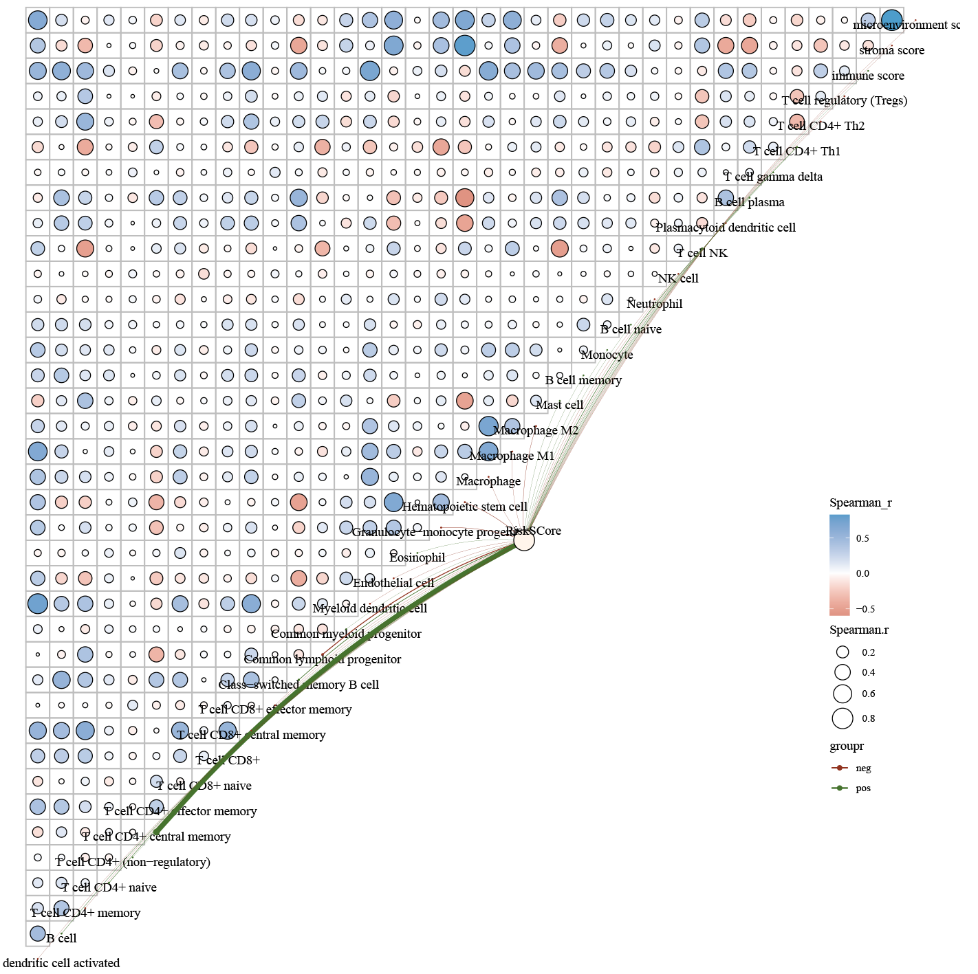
**

**Figure S2: Correlation of risk scores with immunocyte infiltration based on the xCELL algorithm for the PFI prognostic model.**

**
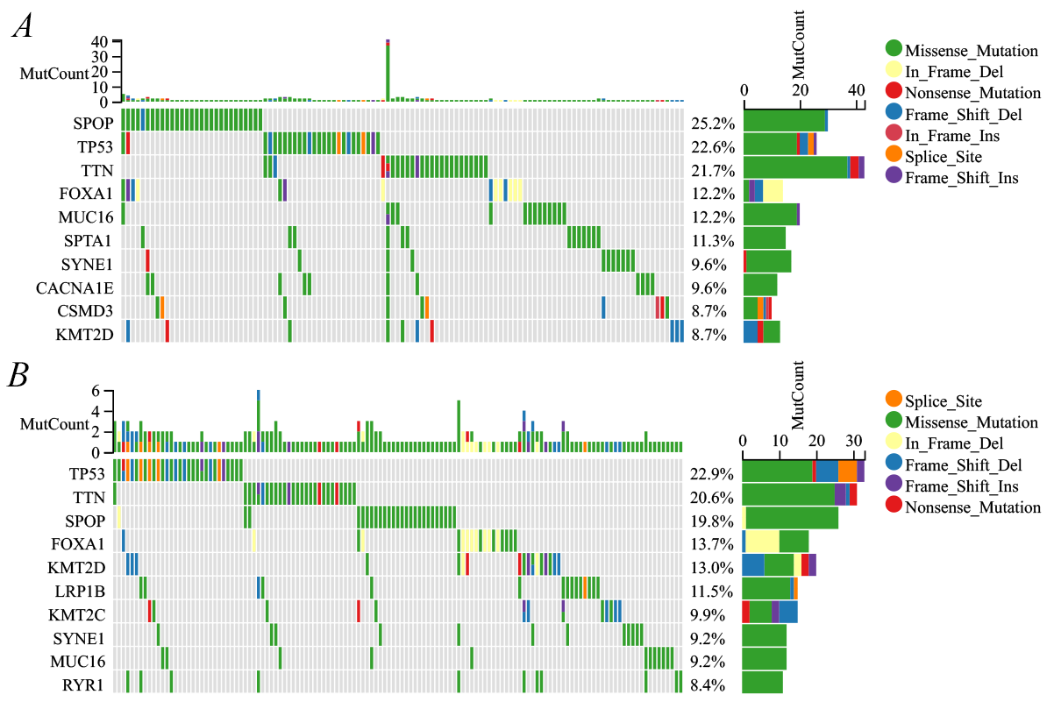
**

**Figure S3: Identification of the top 10 mutated genes under different cluster subgroups**. Mutations under low-risk (A) and high-risk (B) subgroups.

**
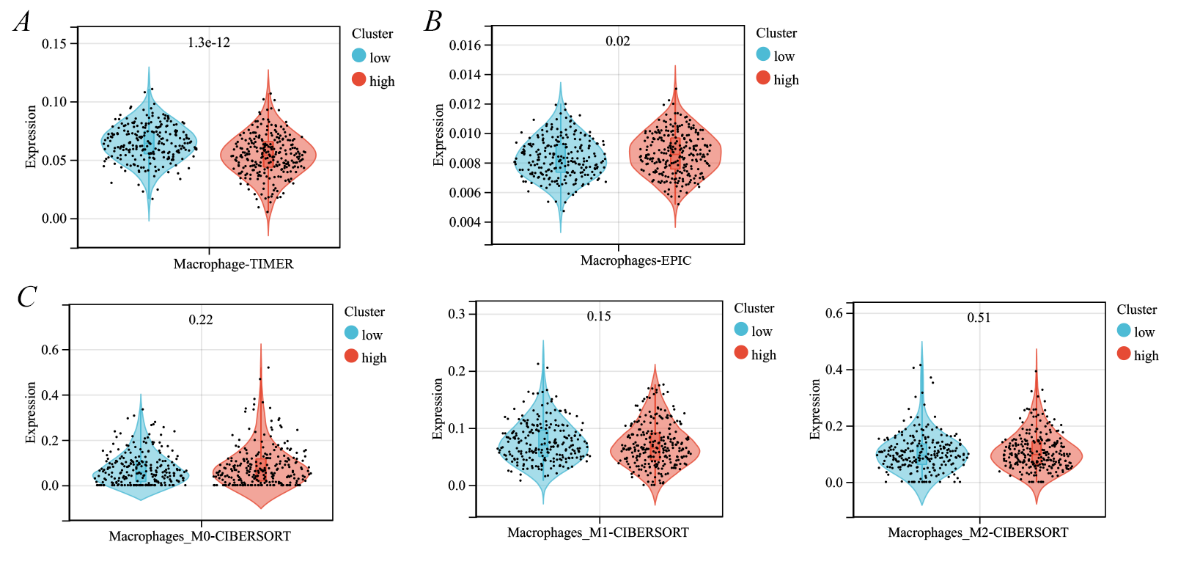
**

**Figure S4: Identification of macrophage expression differences under different cluster subgroups based on multiple algorithms.** (A) TIMER; (B) EPIC; (C) CIBERSORT.

**Tables**

| Characteristics | Low expression of ERLIN2 | High expression of ERLIN2 | P value | Low expression of CDK5RAP3 | High expression of CDK5RAP3 | P value |
| --- | --- | --- | --- | --- | --- | --- |
| n | 250 | 251 |  | 250 | 251 |  |
| Pathologic T stage, n (%) |  |  | 0.058 |  |  | 0.148 |
| T2 | 82 (16.6%) | 107 (21.7%) |  | 104 (21.1%) | 85 (17.2%) |  |
| T3 | 160 (32.4%) | 134 (27.1%) |  | 138 (27.9%) | 156 (31.6%) |  |
| T4 | 5 (1%) | 6 (1.2%) |  | 4 (0.8%) | 7 (1.4%) |  |
| Pathologic N stage, n (%) |  |  | 0.023 |  |  | < 0.001 |
| N0 | 164 (38.3%) | 184 (43%) |  | 180 (42.1%) | 168 (39.3%) |  |
| N1 | 49 (11.4%) | 31 (7.2%) |  | 25 (5.8%) | 55 (12.9%) |  |
| Clinical M stage, n (%) |  |  | 0.253 |  |  | 0.259 |
| M0 | 229 (49.8%) | 228 (49.6%) |  | 226 (49.1%) | 231 (50.2%) |  |
| M1 | 3 (0.7%) | 0 (0%) |  | 0 (0%) | 3 (0.7%) |  |
| Primary therapy outcome, n (%) |  |  | < 0.001 |  |  | 0.001 |
| PD | 17 (3.9%) | 12 (2.7%) |  | 8 (1.8%) | 21 (4.8%) |  |
| SD | 21 (4.8%) | 9 (2%) |  | 8 (1.8%) | 22 (5%) |  |
| PR | 28 (6.4%) | 12 (2.7%) |  | 17 (3.9%) | 23 (5.2%) |  |
| CR | 146 (33.2%) | 195 (44.3%) |  | 185 (42%) | 156 (35.5%) |  |
| Age, n (%) |  |  | 0.065 |  |  | 0.503 |
| <= 60 | 102 (20.4%) | 123 (24.6%) |  | 116 (23.2%) | 109 (21.8%) |  |
| > 60 | 148 (29.5%) | 128 (25.5%) |  | 134 (26.7%) | 142 (28.3%) |  |
| Residual tumor, n (%) |  |  | 0.027 |  |  | 0.032 |
| R0 | 141 (30%) | 175 (37.2%) |  | 170 (36.2%) | 146 (31.1%) |  |
| R1 | 86 (18.3%) | 63 (13.4%) |  | 61 (13%) | 88 (18.7%) |  |
| R2 | 3 (0.6%) | 2 (0.4%) |  | 2 (0.4%) | 3 (0.6%) |  |
| PSA(ng/ml), n (%) |  |  | 0.265 |  |  | 0.938 |
| < 4 | 201 (45.3%) | 216 (48.6%) |  | 204 (45.9%) | 213 (48%) |  |
| >= 4 | 16 (3.6%) | 11 (2.5%) |  | 13 (2.9%) | 14 (3.2%) |  |
| Gleason score, n (%) |  |  | 0.157 |  |  | < 0.001 |
| 6 | 23 (4.6%) | 23 (4.6%) |  | 24 (4.8%) | 22 (4.4%) |  |
| 7 | 111 (22.2%) | 137 (27.3%) |  | 150 (29.9%) | 98 (19.6%) |  |
| 8 | 38 (7.6%) | 27 (5.4%) |  | 18 (3.6%) | 47 (9.4%) |  |
| 9 | 75 (15%) | 63 (12.6%) |  | 57 (11.4%) | 81 (16.2%) |  |
| 10 | 3 (0.6%) | 1 (0.2%) |  | 1 (0.2%) | 3 (0.6%) |  |
| OS event, n (%) |  |  | 0.025 |  |  | 0.341 |
| Alive | 241 (48.1%) | 250 (49.9%) |  | 247 (49.3%) | 244 (48.7%) |  |
| Dead | 9 (1.8%) | 1 (0.2%) |  | 3 (0.6%) | 7 (1.4%) |  |
| DSS event, n (%) |  |  | 0.070 |  |  | 0.371 |
| No | 243 (48.7%) | 251 (50.3%) |  | 248 (49.7%) | 246 (49.3%) |  |
| Yes | 5 (1%) | 0 (0%) |  | 1 (0.2%) | 4 (0.8%) |  |
| PFI event, n (%) |  |  | 0.021 |  |  | < 0.001 |
| No | 193 (38.5%) | 214 (42.7%) |  | 219 (43.7%) | 188 (37.5%) |  |
| Yes | 57 (11.4%) | 37 (7.4%) |  | 31 (6.2%) | 63 (12.6%) |  |

**Table S1: The information table of clinic-pathological features associated with the genes in TCGA-PRAD.**

|  | **Antibody id** | **Sex** | **Age** | **Patient id** | **Grade** |
| --- | --- | --- | --- | --- | --- |
| 1-ERLIN2-normal | HPA002025 | Male | 60 | 2098 |  |
| 1-ERLIN2-PRAD |  | Male | 67 | 689 | High grade |
| 2-ERLIN2-normal | CAB014894 | Male | 51 | 2053 |  |
| 2-ERLIN2-PRAD |  | Male | 53 | 3557 | Low grade |
| 1-CDK5RAP3-normal | HPA022141 | Male | 76 | 2932 |  |
| 1-CDK5RAP3-PRAD |  | Male | 69 | 3580 | Low grade |
| 2-CDK5RAP3-normal | HPA022882 | Male | 76 | 2932 |  |
| 2-CDK5RAP3-PRAD |  | Male | 69 | 3580 | Low grade |
| 3-CDK5RAP3-normal | HPA027883 | Male | 60 | 2098 |  |
| 3-CDK5RAP3-PRAD |  | Male | 58 | 3303 | High grade |

**Table S2: Basic information on immunohistochemistry patients in the HPA database.**
